# Peripheral Osteoma of the Maxillary Sinus in a Patient Planned for Sinus Augmentation

**DOI:** 10.7759/cureus.93482

**Published:** 2025-09-29

**Authors:** Nikolaos Shinas, Fouad Siriani, Vasileios Zisis, Jeremy Kernitsky, Dhurata Shosho, Athanasios Poulopoulos, Deeba Kashtwari

**Affiliations:** 1 Oral and Maxillofacial Radiology, Boston University Henry M. Goldman School of Dental Medicine, Boston, USA; 2 Oral Medicine/Pathology, European University Cyprus, Nicosia, CYP; 3 Oral Medicine/Pathology, Aristotle University of Thessaloniki, Thessaloniki, GRC; 4 Periodontology, Boston University Henry M. Goldman School of Dental Medicine, Boston, USA

**Keywords:** maxilla atrophy, oral defects, oral surgeries, periodontology implantology, peripheral osteoma, sinus lift

## Abstract

Osteomas are slow-growing benign tumors that almost exclusively occur in the craniofacial region and more often in the ethmoid air cells. When they occasionally occur in the maxillary sinuses, they can interfere with dental procedures like sinus augmentation. This study presents the case of a 59-year-old male patient who was treated for sinus augmentation of the left maxillary sinus. During the treatment planning phase, a peripheral osteoma was identified with cone beam computed tomography (CBCT) as an incidental finding without any clinical signs or symptoms. The sinus augmentation procedure was accomplished, and the patient underwent follow-up to confirm no changes in the osteoma. Peripheral osteomas of the head and neck region are usually described radiographically as well-defined and well-circumscribed without clinical signs. Their presence can sometimes interfere with dental treatment, complicating the surgical procedure and even compromising results. Due to their benign and slow-growing nature, and based on their location, size, and clinical relevance, they may not require treatment; however, follow-up is recommended. Peripheral osteomas constitute one of the early findings of familial adenomatous polyposis (Gardner syndrome). This rare autosomal dominant disease is characterized by intestinal polyps and extra-intestinal features, like multiple osteomas and soft-tissue tumors. The early detection of such lesions in the maxillofacial region can lead to timely diagnosis and subsequently improve the prognosis of the patient.

## Introduction

Osteomas are slow-growing benign tumors without clear pathogenesis and are almost exclusive to craniofacial bones [1]. Based on clinical characteristics and location, osteomas are classified as central, when arising from the endosteum, peripheral, when arising from the periosteum, and extraosseous, when occurring in soft tissues [2-4]. Histologically, they can be classified into three categories based on their internal structure. Solid or ivory osteomas are comprised mainly of mature lamellar bone with minimal marrow spaces and a very minor fibrous component. Cancellous or mature osteomas are composed mainly of cancellous bone with bone marrow surrounded by a cortical bone margin and mixed types, which include components from the two main types [1,5].

Osteomas of the paranasal sinuses have a prevalence of approximately 3% and the majority of them involve the frontal sinus in 58-68% of the cases, followed by the ethmoid air cells and the maxillary sinuses (20% of all cases), and finally the sphenoid sinuses, which are rarely involved. Most of these tumors tend to be asymptomatic, with only 4-10% of the cases producing clinical symptoms; however, multiple osteomas of the maxillofacial region can be associated with Gardner syndrome [6]. 

Sinus augmentation is a surgical procedure used to increase the bone volume in the atrophic posterior maxilla to support the placement of dental implants [7]. The first sinus augmentation was performed by Tatum in 1976, which involved placement of autogenous bone or bone substitute, healing for six months, and finally implant placement, and was first described by Boyne and James in 1980 [8]. Solitary osteomas in the maxillary sinuses are often found as incidental findings [9], and pre-existing sinus disease can be associated with post-surgical complications or compromised results in patients who are planned for sinus augmentation procedures [10]. This study presents a case planned for sinus augmentation and subsequent implant placement with an osteoma in the left maxillary sinus.

## Case presentation

In 2023, a 59-year-old male patient was referred to the Oral and Maxillofacial Radiology (OMR) Clinic of Boston University Henry M. Goldman School of Dental Medicine from the Department of Periodontology for a cone beam computed tomography (CBCT) of both mandibular and maxillary arches. The patient was planned for sinus augmentation of the left maxillary sinus to increase the height of the atrophied alveolar ridge to facilitate implant placement. No clinical findings were reported by the patient during the time of acquisition. 

**Figure 1:**
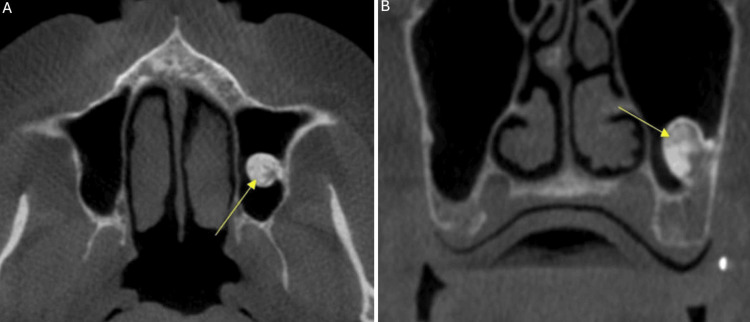
(A) Axial view at the level of the inferior nasal conchae, area of the left maxillary sinus; CBCT acquired in 2023. (B) Coronal view at the level of the missing first and second molars, area of the left maxillary sinus; CBCT acquired in 2023. The yellow arrows show the well-defined non-uniform high-density entity under investigation. CBCT: cone-beam computed tomography

The CBCT examination was performed with i-CAT™ FLX V17 (DEXIS LLC, Pennsylvania, United States) with exposure settings set at 120 kVp, 5 mA, and 3.708 seconds, and a volume of 16x10 cm was acquired. Radiographic interpretation of the scan concluded that the left maxillary sinus was well-delineated with dimensions within the range of normal. Mild thickening of the mucosa was detected, a finding consistent with mild mucositis. On the floor of the left maxillary sinus, a well-defined non-uniform high-density entity was visualized. The shape of the entity was pedunculated, and it was attached to the posterior-lateral floor of the sinus. No radiographic evidence of effect on adjacent structures was detected (Figure 1).

**Figure 2:**
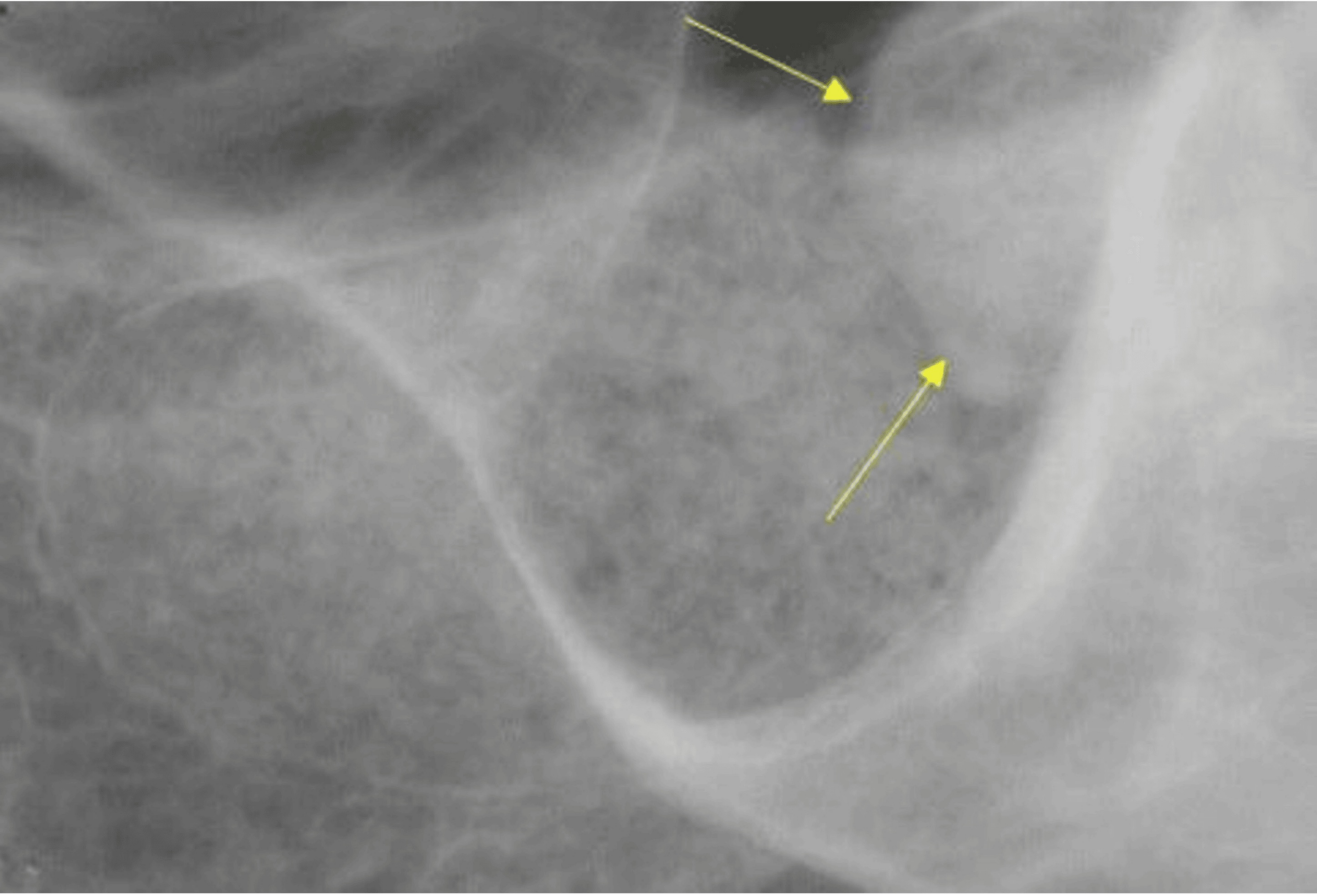
Intraoral periapical (PA) radiograph of the area of missing #14 and #15, acquired in 2024 at the one-month follow-up after the sinus augmentation procedure. The yellow arrows show the high-density entity under investigation.

Radiographic findings of the entity in the left maxillary sinus were consistent with a slow-growing benign tumoral process, most likely a peripheral osteoma. The general differential diagnosis for an osteoma in the paranasal sinus includes other benign bone tumors like osteoblastoma, inflammatory lesions such as chronic sinusitis, and fibro-osseous lesions like fibrous dysplasia. Other conditions to consider are mucocele/pneumatocele, sinonasal undifferentiated carcinoma, and Gardner syndrome if multiple osteomas are present. Definitive diagnosis relies on imaging, such as a CT scan, which typically shows osteomas as well-circumscribed, hyperdense, bony masses. However, in this case, the diagnosis was relatively straightforward; no other differential diagnoses were considered. After the scan was acquired, the patient was dismissed, and approximately 10 months after the initial CBCT evaluation, the sinus augmentation was performed in 2024. One month after the procedure, an intraoral periapical radiograph (PA) was acquired to assess the healing process (Figure 2).

**Figure 3:**
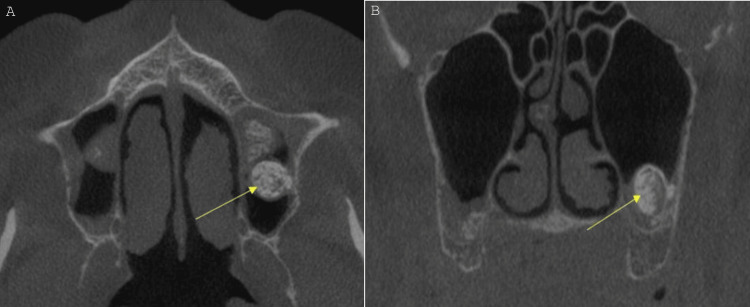
(A) Axial view at the level of the inferior nasal conchae, area of the left maxillary sinus. The entity in the left sinus has been unchanged. (B) Coronal view at the level of the missing first and second molars, area of the left maxillary sinus. CBCT acquired in 2024, seven months after the sinus augmentation. The yellow arrows show the well-defined non-uniform high-density entity under investigation. CBCT: cone-beam computed tomography

Finally, seven months after the sinus augmentation, a second CBCT was performed to assess bone height and width, and to confirm that no changes took place in regard to the osteoma. The radiographic evaluation of the second scan revealed that the entity had been stable in size with no changes (Figure 3).

**Figure 4:**
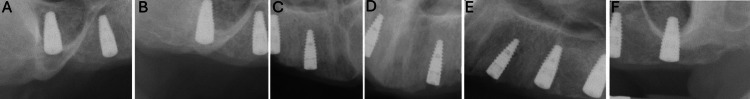
Periapical radiographs of the maxillary implants two months after the placement. No radiographic evidence of associated pathosis noted. A,B,C: Implants in the first quadrant. D,E,F: Implants in the second quadrant.

Subsequently, implants were placed in the maxilla in 2025, and PAs were used to assess the placement two months after the procedure (Figure 4).

## Discussion

Peripheral osteomas in the head and neck region are most commonly identified on radiographs as well-circumscribed, radiopaque masses, either oval or round in shape, with well-defined and smooth margins that lack any perilesional halo, helping to distinguish them from other osseous lesions [1,11]. These lesions manifest with hard consistency, either pedunculated and attached to the cortical plates by a stalk or with a broad base that connects directly to the cortical bone [12]. The maxillofacial osteomas are radiopaque and may protrude from the periphery of the jawbone, either on the lingual, palatal, or, less commonly, buccal surfaces, making them relatively straightforward to recognize and diagnose on conventional imaging [11,12]. They exhibit a bone density similar to that of normal bone, without any surrounding bone destruction, and are generally unilateral, which further aids in their differentiation from more aggressive bone pathologies or conditions such as idiopathic osteosclerosis [1,11]. The use of advanced imaging techniques, particularly CT scans with three-dimensional (3D) reconstruction, provides superior resolution and allows for more accurate localization and characterization of the osteoma, allowing for precise surgical planning when necessary [1]. Given these radiographic features, the careful selection of imaging technique ensures the diagnosis and the subsequent management, ruling out syndromic associations [12].

Most craniofacial osteomas are asymptomatic, which explains why they are frequently identified as incidental findings rather than through clinical symptoms or physical examination [13]. The prevalence of these asymptomatic lesions varies depending on the imaging technique; on CBCT examinations, osteomas were detected in 7% of 499 images analyzed [14], on CT examinations, craniofacial osteomas account for approximately 3% of all incidental findings, while their detection rate drops to about 1% on plain radiographs [15]. This discrepancy highlights the superior sensitivity of CT imaging in detecting small or otherwise inconspicuous lesions.

The asymptomatic nature and slow progression of most peripheral osteomas often justify a conservative management strategy, emphasizing observation and routine follow-up rather than immediate intervention [15,16]. Radiological features, typically presenting as well-circumscribed, oval radiopaque masses attached to the cortex, play a pivotal role in the initial assessment and can help distinguish these lesions from other entities; however, definitive diagnosis hinges on histological examination due to the overlap in radiographic appearance with other jaw lesions [15,17].

A crucial point in diagnosis is the presence of multiple lesions, a finding associated with familial adenomatous polyposis or Gardner syndrome, a rare autosomal dominant disease. The syndrome is characterized by intestinal polyps and extra-intestinal features, such as multiple osteomas and soft tissue tumors, with osteomas being one of the early syndromal manifestations. The clinical oral and radiographic examination of such patients can play a pivotal role in diagnosis, especially when considering the increased chance of malignant transformation due to polyp dysplasia with a mean age of diagnosis of 40 years [18,19].

The lesion’s size, location, often in the mandible, and potential for causing facial asymmetry, functional impairment, or cosmetic concerns further influence clinical decision-making. Although surgical removal is generally reserved for symptomatic cases or those exhibiting significant growth or aesthetic impact, the absence of reported complications or recurrences in most cases following excision supports the safety of the surgical approach [16]. Nevertheless, the rare possibility of recurrence, even years after surgery, supports the necessity for periodic clinical and radiographic monitoring, regardless of initial presentation [18].

## Conclusions

Peripheral osteomas are benign and slow-growing tumors that almost exclusively occur in the maxillofacial region. When occurring in the maxillary sinuses, they can interfere with dental procedures, like sinus augmentation. Radiographically, osteomas are well-defined, radiopaque entities that are usually identified as incidental findings, and clinically, they are often asymptomatic with no effects on adjacent structures, thus requiring no treatment. Like in the case presented in this report, not all osteomas require surgical excision and immediate treatment; however, close monitoring and long-term follow-up are crucial to ensure no changes in the shape and size. Finally, it is imperative for the clinician to identify such lesions, especially when they occur in clusters and/or multiple regions, since multiple osteomas of the craniofacial complex can be associated with familial adenomatous polyposis (Gardner syndrome). This rare disease can present with intestinal and extra-intestinal signs and symptoms; however, the presence of multiple osteomas is one of the earliest and crucial manifestations that can aid in diagnosis. This is one of the few conditions where early diagnosis has a huge impact on prognosis, and dentists may be the first to detect a life-threatening syndrome and refer for medical care.
